# Indents

**Published:** 1906-03-15

**Authors:** 


### INDENTS.

☞ **Brief Articles of Technical or Scientific Value will receive notice and comment. Contributions invited.**

**Alveolar Abscess Symptoms.** Every case of alveolar abscess begins with an inflammation of the apical portion of the peridental membrane following immediately or more or less remotely the death of the pulp of a tooth. If acute, there is severe pain and soreness of the tooth. In most cases this is accompanied by a lymphangitis in which the lymphatic glands of the floor of the mouth, the submaxilliary lymph glands and those of the angles of the neck are more or less involved. Generally pus forms about the apex of the tooth promptly and more or less absorption of bone in the immediate neighborhood takes place quickly. During this process a sharp rise of temperature occurs, often preceded in the severe cases by a chill. The severity of these symptoms gives indications of the probable vile results that are to follow. It is well at this time, if the symptoms are severe, to look carefully to the general condition of the patient, particularly to see that the circulation is good and that the alimentary tract is in good condition. When there is considerable severity the bowels should be cleared with saline cathartic or some form of catharsis which gives large, watery stools. A fever of more than 103 degrees betokens danger in some direction and 105 degrees or 106 degrees becomes in itself dangerous unless its duration be very short.—Dr. Black, in *Northwestern.*

**Fees and Ethics.** Every real service rendered ought to exact a fee that is commensurate, and one who does not make this a business rule is not persuing a safe business method. While a contraiy practice may not be classified unethical it has a cheapening tendency and may be unfair to one's confrerers.

It goes without saying that the most perfect service or article is the cheapest; it has always been so; it always will be. Therefore, my suggestion is, address yourselves to the highest methods attainable, that the results may be yours to give your patrons. Get good fees for your services, for without good fees there can be no leisure; without leisure there is no time for thought, and without thought no progress. They who stand in the way of professional progress are the professional cut-throats, because of the violation of every ethical principle in practice.

Competition spurs us on for the mastery, but we cannot lose sight of the fact that we must dignify our occupation. The preparation for professional pursuits requires valuable time, and a large outlay in money. One who enters upon professional study to-day so understands it, and when his course is finished, his diploma is given as evidence of a high calling, with a great responsibility attaching to it, namely, Usefulness, Self-respect, Dignity, Honor and Progress.

Let it be understood that we contend that no professional man can pursue a cut-throat policy in practice, and at the same time maintain an ethical attitude toward his clientele and competitors. Only striving to occupy the higher ground where there is room for all is permissible, and the only woik worthy of engaging our attention.—W. H. Chilson, in *Review.*

**Diffused Alveolar Abscess.**

If the pus, after passing through the bone, spreads between the bone and the periosteum, there will be presented a soft, flabby swelling without definite margins, easily found by the fingers. In this form if an examination is made with the probe after the discharge of the pus, the bone will be found denuded of periosteum over a considerable surface. This is the most dangerous form of alveolar abscess. Wherever there is serious damage by necrosis of bone, prolongation of suppuration, or imminent danger to life quickly occurs, it is from this form of the abscess as a rule.

The particular location of the tooth is not important. The grave results arise from the pointing of the pus in such a way as to begin spreading between the bone and its periosteum. In this case the pus will spread to the limits of easily raised periosteum and often will quickly travel considerable distance. For safety such cases require immediate radical treatment by drainage as soon as they are discovered. The pus should be discharged by a free incision, the pus cavity well washed with a mild antiseptic solution, and then a sufficiently large tent of plain antiseptic gauze, or of cotton, should be placed well into the opening to insure full and complete drainage. This tent should be prepared by being dipped in 95 percent carbolic acid and then dried out as thoroughly as possible by squeezing between thick folds of a towel, or a sufficient amount of absorbent cotton. The small amount of carbolic acid retained in the gauze is sufficient to cauterize the lips of the incision without injury to the surrounding tissue, makes the opening freer than it would otherwise be, promotes the egres of pus and at the same time presents more of a barrier to the ingress of other infectious material. Dr. Black, in *Northwestern.*

**Useful Hints.** The "Artists Dividers" are handy for getting the length or width of teeth in or out of the mouth, and a pair of delicate ones that can be set or adjusted at pleasure should be in every outfit. They will save much time and money in secuiing the pioper width ot material for ciowns, bands, etc.

When the screw mandrel wears and gets loose so that it will not retain the stone or disk, place the mandral shank on the anvil and strike it a sharp blow with a small hammer, this will flatten the hole and make the screw thread bind tightly.

It is often difficult to securely wax together a broken plate, especially a lower partial. The attempt to hold the broken pieces in one hand while you attach them with the other makes you wish you had three or four hands. If a strip of wax, such as the dental depots use to attach a set of teeth to, be softened and laid on a flat, unyiedling surface, a piece of glass for instance, the broken plate may be pressed teeth down, into the wax, using both hands to hold the pieces in proper position. When you let go, they will remain in place and you are free to examine carefully and wax together.

When packing a plate many lay a napkin over a dish of hot water, allowing the steam to soften the vulcanite rubber. The napkin often sags down into the water or slips off and causes considerable annoyance. By stretching a single thickness over a wooden embroidery hoop you have an ideal contrivance. The rubber will not fall off or stick to the napkin and you will be pleased with the result.

In giving the final polish to rubber plates, I find that a polishing wheel made of about twenty-five thicknesses of unbleached muslin gives a better finish than chamois skin or a soft brush wheel. The edges of the circles of cloth become frayed and do not scratch. I can get a finer polish with this kind of polishing wheel than with anything else I know of.—A. C. Willman, in *Review*.

**Sanitary Bridge-work.** We are not practicing oral hygiene in many cases where we use the stationary bridge with either the saddle or the so-called self-cleansing space. But we are practicing oral pollution and infection. In my opinion the only approximation to sanitary conditions in fixed bridgework is one or the other of the following forms: The solid gold bridge where occlusal surface only of dummies is supplied, and these should have smooth, well-polished convex surfaces on side next to gum and with ample space for brushing and clearing of food. This form of bridge is limited in its application and is seldom used other than to supply lower posterior teeth. The other form of fixed bridge that possesses sanitary possibilities is the all-porcelain bridge constructed with narrow, well-fitted saddle and in very carefully selected cases.

That the fixed bridge has no place I am not prepared to say, but that the removable bridge has a wider field, with greater possibilities and many advantages, I am thoroughly convinced. The advantages of the removable over the fixed bridge is incomparably a more sanitary denture, greater possibilities of restoration of the lost tissues and more natural and artistic results. Repairs are made simple and easy, and any subsequent repairs upon adjoining teeth are simplified and less destruction of natural teeth is required for abutments. Another advantage of no small importance is the fact that good, serviceable, as well as very artistic bridges may be made with vulcanite at a moderate cost.

The ideal material for this class of work is porcelain, and, where cost is not a consideration, it may be almost universally used. Next in order of preference is the gold saddle, with porcelain teeth soldered thereto, and usually some of the manufactured porcelain crowns are most suitable, but good results may be obtained with ordinary rubber teeth and vulcanite base. Several systems of anchorage have been devised, and while they differ in some 1espects they are in the main all quite similar.—F. E. Roach, in *Review*.

**Edema from Infection.** Infection that induce edema occurs frequently, both in connection with alveolar abscess and without. It seems to be much less important when connected with abscess than when standing alone, for the reason that the serum more readily escapes from the tissues. The charactistics when alone are broad local swellings without suppuration and accompanied by considerable pain from the distention of the tissue and the inflammatory movement. When standing alone I have most often seen it occur from some slight prick or puncture; but it has occurred a number of times in extraction wounds. I have met with two cases of imminent dange1 of death from suffocation from the edematous swelling of the glottis and associate parts, primarily caused by infections occuring in the mouth. One of these was from infection following the extraction of a tooth, and the other from the prick of an instıument in the floor of the mouth.

In the extraction case I was called forty-eight hours after the swelling began. The swelling of the face and throat was enormous. The patient was struggling for breath and cyanosis was very pronounced, the lips and mucous membranes appearing distinctly bluish. A brisk saline cathartic had been given by the physician and to assist more directly in removing the accumulation of serum, I pushed my knife through the tissues in various directions and placed the feet in hot water. In the other case the danger to life was not so imminent, but the swelling on the floor of the mouth was so great that the raphe under the tongue upon which the submaxilliary and sublingual glands open was protruded oveı the lower teeth. The mouth could not be closed and there was much difficulty in breathing. This was treated similarly to the other case and with good results. In both these cases I made very free use of the knife purely for the purpose of giving opportunity for the escape of the serum. Such cases are liable to be confounded with alveolar abscess.—Dr. Black, in *Northwestern*.

**Cuspid or Canine.** In the September *Dental Cosmos* appeared the report and discussion thereon, of the committee on Dental Nomenclature, read before Section IX of the fourth International Dental Congress. We notice by this report the chairman of the committee and one of our leading dental editors, very strongly advocated the term canine and not cuspid for the third tooth from the median line in the dental arch. Because of the importance we know the profession will attach to the opinion of these men we are constrained to disapprove of their conclusions and give our reasons for thinking otherwise.

Science is classified knowledge. All sciences have a system of names or nomenclature. The nearer the names conform to a scientific basis, the nearer it is to perfection. In naming an object to-day a descriptive word or phrase is usually chosen; but often it is not possible to give a complete description in the name, therefor the most prominent feature or features only may be included. Certainly the most prominent feature of the cuspid tooth is the pointed form, which is recognized by both, the educated and uneducated mind. Canine seems to me far-fetched because it is only the mind having some knowledge of comparative anatomy that can see any resemblance between the pointed tooth in man and a dog. Science requires that scientific terms be expressed in scientific language which cuspid does, and without an offensive inference included.

The teeth are divided into three groups: incisors, molars and cuspid-bicuspid. The naming of the first two classes indicate their function and the third class their form. To subdivide the cuspid-bicuspid class only complicates the nomenclature. Certainly there is no reason for selecting a dog to furnish a name for this useful member of our dental armament. It seems to me if it were necessary to honor a carnivora, that it would have been more grand to have named it after the King of Beasts; or if it was necessary to call attention to our beast-like nature, it would have been more appropriate to have named it for our sometimes called progenitor.—Dr. G. H. Wilson, in *Dentist's Magazine.*

**Rubber Dam Adjustment.** RULE.—*If after the adjustment of the rubber dam the operative field is not one of greatest convenience, do not go on with the operation; remove the dam and so re-adjust it that the greatest convenience exists for the patient as well as for the operator.*

It goes without saying that no man living can make as thorough operations in an inconvenient environment as in a convenient one.

I have no occasion to change my already expressed ideas regarding cleanliness. The tissues against which the dam is to rest should be rendered as surgically clean as possible through washing the parts and teeth with warm, sterilized water. Any debris in the interproximal space or on the crowns of the teeth should be removed prior to adjusting the rubber dam. The dam should be cleaned by washing it with soap and water, and after it has been dried it should be rolled in antiseptic French chalk. The lips, if cracked, and any sores on the face should always be covered with a salve of some kind, depending wholly upon the conditions. Vaseline, cold cream and resinol all have their places. Napkins or other means should always be used to protect the face from contact with the dam and the holders used to secure it in place. The use of ligature has been dealt with in other essays. It may be well, however, to say that if the holes in the rubber dam are punched sufficiently far apart, so as to completely fill the mesio-distal width of the interproximal space, moisture will seldom seep in. Therefore, the tying to ligatures, except on the tooth to be operated on and the adjoining one, is unecessary. If the gums are washed with oil of cloves immediately before adjusting the rubber dam, the pain incident to the application of clamps or ligatures is very greatly minimized. There is one other thing which I do not remember seeing in print, and that is a method of securing a clamp to some upper second molars. These teeth are occasionally of such a conical form as to make it somewhat difficult to keep a clamp upon them. If a ligature is tied around the gingival margin several times and securely tied, and then the clamp adjusted right into the thread itself, the clamp will not slip from that tooth.—Dr. E. K. Wedelstadt, in *Dentists Magazine.*

**Atrophied Teeth.** Dr. J. Austin Dunn reports the following case of a girl seventeen years of age, with the following conditions: The incisors and cuspids of both jaws are conical in shape, the surfaces irregular and more or less grooved, and pitted, with very little enamel excepting at the gingival lines, where it is normal. The bicuspids are nearly normal in all respects. The first molars of both jaws have not developed above the gingival lines, having a flat, glassy-brown surface with no sign of a pulp chamber. The mesial third of the second lower molars have developed, but the middle and distal portions are only partly developed and irregular. The second upper molars are normal, excepting the occlusal surfaces, which are flat. The third molars are fully developed, and this can probably be accounted for from the fact that the young lady was in delicate health up to her seventh year, when there was a decided improvement. All of the teeth are brown in color, excepting the third molars, with roots fully developed. The twelve upper teeth have been restored by means of the tube, or cylindrical method, without the least sign of sensitiveness.

The history of this case covers three generations, namely, the young lady's grandfather, father and brother, also two sisters of the father and some of their children. Her brother has three children, of which two have normally developed teeth, but the crowns of the deciduous teeth of the girl baby, two years of age, have not developed, being flat, and even with the gums and brown in color.

A married sister has perfectly formed teeth, also her children. In this connection I wish to ask why should this child be the only one in the family to develop a normal set of teeth? My answer is, that she is "like her mother in every way," and I believe that the mother's personality was so strong that it dominated during gestation to such an extent that it overcame even a strong hereditary tendency from the paternal side.

This case is similar to two reported in the American System of Dentistry, vol. 3, page 415, by Dr. W. C. Barrett, of Buffalo, and Dr. D. B. Freeman, of Chicago. I find by comparison that this case is more extensive than those just referred to, and besides we have both heredity and atrophy as etiological elements to consider, hence it is difficult to classify this particular case.—*Review.*

